# Circulating microRNA-208 family as early diagnostic biomarkers for acute myocardial infarction: A meta-analysis

**DOI:** 10.1097/MD.0000000000027779

**Published:** 2021-12-23

**Authors:** Jia Wang, Liwenjing Xu, Lu Tian, Qiyu Sun

**Affiliations:** Department of Clinical Laboratory, Affiliated Hospital of Chengde Medical University, Chengde, China.

**Keywords:** acute myocardial infarction, biomarkers, meta-analysis, miRNA-208

## Abstract

**Objective:**

Many recent studies have demonstrated that serum miRNA-208 (miR-208) could be a powerful biomarker in the early diagnosis of acute myocardial infarction (AMI). However, the result of previous studies was not accurate due to the small sample sizes and controversial issues. Therefore, this study was performed to investigate the relationship between the expression levels of miR-208 and AMI.

**Materials and methods:**

According to the inclusion and exclusion criteria, a preliminary literature search was performed. The study was based on articles published in PubMed, Embase, Cochrane databases before September 30, 2019. Two staff members extracted data from the included articles for meta-analysis. These data were analyzed for sensitivity, specificity, diagnostic odds ratio, and summary receiver operator curve (SROC) analyses.

**Results:**

This study included 13 pieces of literature, which contains 1703 patients with AMI and 1589 controls. The main results of our meta-analysis were as follows: The pool sensitivity and specificity of miR-208 for diagnosing AMI was 83% and 97%. The area under the SROC curve (AUC) was 93%. Mir-208 had a highly effective diagnostic capacity to distinguish AMI from chest pain patients with an AUC of 93%.

**Conclusions:**

The results showed that circulating miR-208 was a reliable biomarker both for diagnosting ST-elevation myocardial infarction (STEMI) and non-ST elevation myocardial infarction (NSTEMI). MiR-208 was sufficient to distinguish AMI patients with chest pain from healthy controls.

## 1 Introduction

At present, coronary heart disease has become one of the leading causes of mortality in China, among which acute myocardial infarction (AMI) takes the highest proportion.^[1]^ Cardiac markers commonly used in the clinical diagnosis of AMI include creatine kinase-MB (CK-MB), cardiac troponin T (cTnT), and myoglobin, etc.^[2]^ Previous studies showed that the significant levels of cTnT were identified around 6 hours,^[3]^ and high- sensitivity cTnT could only be detected within 3 to 4 hours after the myocardial infarction.^[4]^

Therefore, a highly sensitive, specific, early detected biomarker to reliably exclude AMI or diagnose AMI immediately is needed.^[5,6]^ Recent studies have shown that cardiovascular diseases can cause significant changes in the expression level of specific MicroRNAs (miRNAs) in the body. Therefore, the detection of particular miRNAs in body fluids can play an essential role in diagnosing and preventing cardiovascular diseases.^[7]^ MiRNAs are a class of nonprotein encoded small RNAs that widely exist in eukaryotes and have a length of 21 to 25 nucleotides.^[8]^ They are highly stable in blood circulation and can regulate gene expression in a sequence-specific manner. They play an essential role in development, apoptosis, metabolism of the human body, and human diseases. The physiological and pathological regulation mechanism of miRNA has been highly valued in recent years.^[9]^ A crucial role of many miRNAs in the development and function of heart and blood vessels in the human body was demonstrated in previous sequence-, microarray-, and other array-based profiling studies.^[10]^ Many studies had revealed that detection of the microRNA expression in the blood can be served for assaying the biological substances and evaluating the prognosis of myocardial infarction. MiR-208 was expressed in myocardial cells and showed a close association with the development of cardiac diseases, such as myocardial hypertrophy, cardiac fibrosis, myocardial infarction, arrhythmia, and heart failure.^[11]^ In recent years, many articles have reported the diagnostic value of miR-208 for AMI and made great progress,^[12–26]^ although many studies have reported that miR-208 has a certain value for the diagnosis of AMI, the results of some studies were inconsistent. Wang et al^[12]^ reported a higher specificity (100%) and a higher sensitivity (98%) of miR-208 for AMI diagnosis. But Li et al^[13]^ revealed a lower sensitivity of miR-208b (75.8%) and a lower specificity (73.1%). This study aimed to determine the diagnostic and prognostic value of miR-208 by summarizing the last ten years of articles and aimed to reveal the application value of the microRNA-208 family, in the diagnosis of myocardial infarction through meta-analysis. To provide evidence for early clinical diagnosis and medication.

## 2 Materials and methods

### 2.1 Literature retrieval

Institutional review board approval and patient consent were not required due to the nature of this study. A preliminary literature search was performed using PubMed, Embase, Cochrane, and CNKI databases. The search took place from October 2019 to September 2020 for the database's inception. Using the terms “circulating” or “plasma” or “serum” and “microRNA-208” or “miRNA-208” or “miR-208” and “myocardial infarction” or “AMI.” Besides, manual searches of the references included were conducted to prevent the omission of high-quality articles. The search strategy was to search the databases by combining the subject words with natural language terms. Criteria for the inclusion and exclusion of published studies. The pooled results included the sensitivity and specificity of microRNA-208 for AMI. The area under the summary receiver operating characteristic curve (AUC) was used to estimate overall test performance.

### 2.2 Literature inclusion and exclusion criteria

The investigative team developed inclusion and exclusion criteria. The inclusion criteria were:

1. human studies,
2. studies related to circulating miRNAs levels and AMI, and
3. studies that contained enough data to evaluate the diagnostic value of miRNAs in AMI.

Exclusion criteria are as follows:

1. Papers are written in a language other than English,
2. A meta-analysis of review letters reviews posters,
3. Experimental design based on the animal model only,
4. Not associated with acute myocardial infarction.

### 2.3 Quality evaluation

Studies reporting on miR-208 family were included in the meta-analysis and were evaluated according to the Quality Assessment of Diagnostic Accuracy Studies 2 (QUADAS-2) checklist,^[14]^ which was designed to assess the risk of bias and the applicability of studies of diagnostic accuracy. The following 4 key domains were included: patient selection, the index test, the reference standard, and flow and timing. Each was assessed with respect to the risk of bias, and the first 3 domains were assessed with respect to applicability.

### 2.4 Data retrieval

To guarantee the reliability of the results, 2 workers took the required documents. If there was a disagreement regarding a particular article's eligibility for the analysis. It can be resolved by consensus. Research data extraction included the first author, published in the country, race, country of origin, the number of cases and controls, biomarkers, and the indicated biomarker's sensitivity and specificity for AMI diagnosis. We used GetData graphics digitizing software to digitize the graphic data in articles.

### 2.5 Statistical analysis

The overall sensitivity evaluated the diagnostic value of miRNA-208 in patients with AMI. We used STATA (15.0 Stata Corp LP, College, Station, TX) to construct specific positive likelihood ratio (PLR), negative likelihood ratio (NLR), diagnostic advantage ratio (OR), receiver operating characteristic curve, and 95% confidence interval forest plots. Due to the study's assumed heterogeneity, we used the Meta-DiSc 1.4 (XI Cochrane Colloquium, Barcelona, Spain) to obtain the AUC of miR-208 non- ST- elevation myocardial infarction (NSTEMI) and ST- elevation myocardial infarction (STEMI). The random-effect model (Del Simon-Laird method)^[15]^ was used to assess the included study's heterogeneity using *I*^2^. If *I*^2^ > 50% or *P* < .05, metaregression analysis was performed to find the effect of potential heterogeneity in sensitivity and specificity.

## 3 Result

### 3.1 Result of literature retrieval

**Figure 1:**
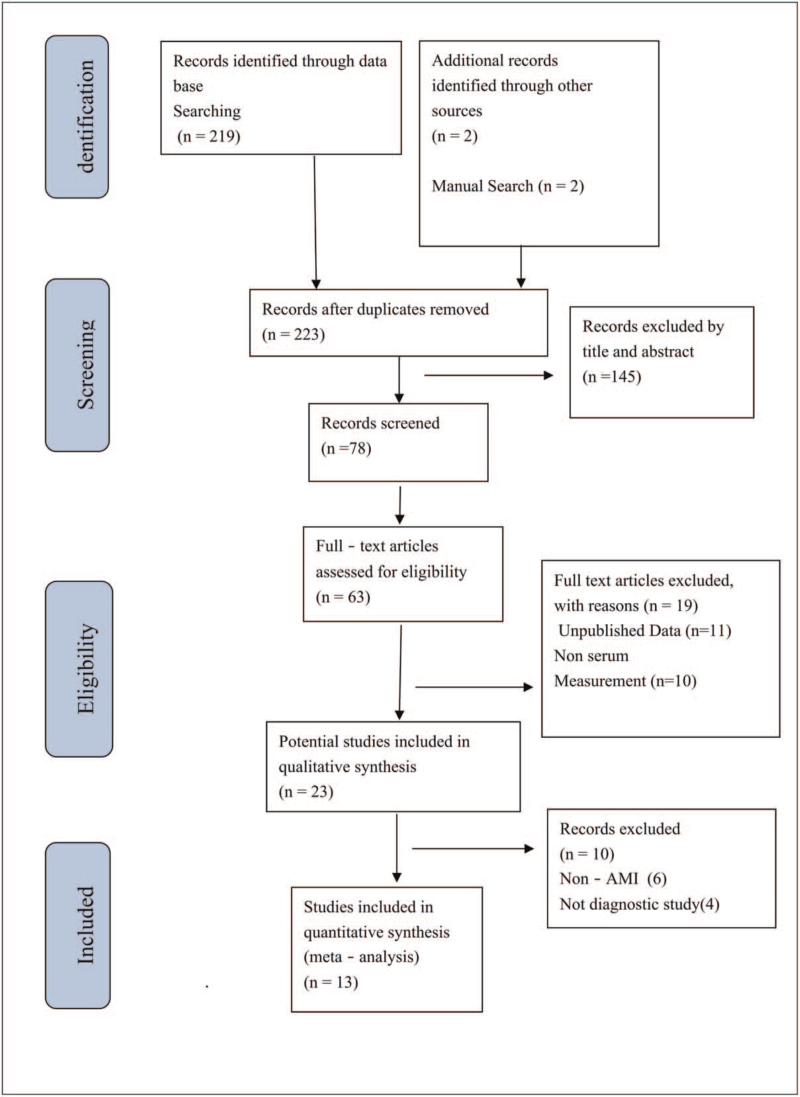
Flow diagram of the literature search process and study inclusion.

The procedure for the included studies was shown in Figure 1. We conducted a preliminary search of miR-208 and found a total of 219 relevant pieces of literature written in English through database searching and other sources. After the initial screening, based on the abstracts, headlines, and article types, only 13 works of literature met the inclusion criteria.^[12,13,16–26]^ Three articles were discarded due to the lack of valid data.^[27–29]^ The necessary information included in the studies was contained in Table 1. The studies were conducted in 5 countries; most of the subjects in the study were East Asians. A total of 3292 patients (1703 patients with AMI and 1589 non-AMI subjects) were included.

**Table 1:** Characteristics of studies included in the systematic review.

References	Country	Specimen	Case (n) / Control (n)	Age (Case)	Methods	miRNAs	Patient characteristics (Control)	Max time from onset until sample acquisition	AUC	Sensitivity	Specificity
Li et al, 2019^[23]^	China	Serum	41/32	62.95 ± 11.04	SYBR	miRNA-208	AMI/Non-AMI ACS	Within 3 h	0.868	70%	97.5%
Agiannitopoulos et al, 2018^[22]^	Greece	Plasma	50/50	62.12 ± 10.99	TaqMan	miRNA-208a	AMI/Healthy	Within 24 h	0.999	98%	100%
Liu et al, 2018^[24]^	China	Plasma	145/30	67	SYBR	miRNA-208	STEMI/Healthy	Within 2–4 h	0.994	90%	100%
Li et al, 2015^[13]^	China	Plasma	87/87	56.93 ± 9.17	SYBR	miRNA-208b	AMI/Healthy	Within 4 h	0.674	59.8%	73.6%
Liu et al, 2015^[26]^	China	Plasma	70/72	64.2 ± 11.2	TaqMan	miRNA-208b	AMI/Healthy	Within 2 h	0.72	65%	90%
Devaux et al, 2015^[21]^	Luxembourg	Plasma	224/931	72 (61 ± 80)	TaqMan	miRNA-208b	AMI/Non-AMI ACS	Within 12 h	0.76	64.7%	80.2%
Gidlof et al, 2013^[20]^	Sweden	Plasma	318/88	64.56 ± 2.7	SYBR	miRNA-208b	AMI/Non-AMI ACS	Within 72 h	0.82	79%	70%
Li et al, 2013^[25]^	China	Plasma	117/100	62.7 ± 11.4	TaqMan	miRNA-208	AMI/Healthy	Within 2 h	0.778	75.8%	73.1%
Li et al, 2013^[19]^	China	Plasma	67/32	63.84 ± 11.17	SYBR	miRNA-208b	AMI/Healthy	Within 12 h	0.89	82.4	99.8%
Devaux et al, 2012^[18]^	Netherlands	Plasma	510/87	62 (0.32–91)	TaqMan	miRNA-208b	AMI/Healthy	Within 12 h	0.9	79.5%	99.8%
Gidlof et al, 2011^[17]^	Sweden	Plasma	9/11	64.56 ± 2.7	SYBR	miRNA-208b	STEMI/Healthy	Within 12 h	1	1	1
Corsten et al, 2010^[16]^	Netherlands	Plasma	32/36	62 ± 13	SYBR	miRNA-208b	AMI/Non-AMI ACS	Within 12 h	0.965	90.6%	94.1%
Wang et al, 2010^[12]^	China	Plasma	33/33	63.5 ± 10.1	TaqMan	miRNA-208	AMI/Non-AMI ACS	Within 12 h	0.999	98%	100%

### 3.2 Quality of the included studies

**Figure 2:**
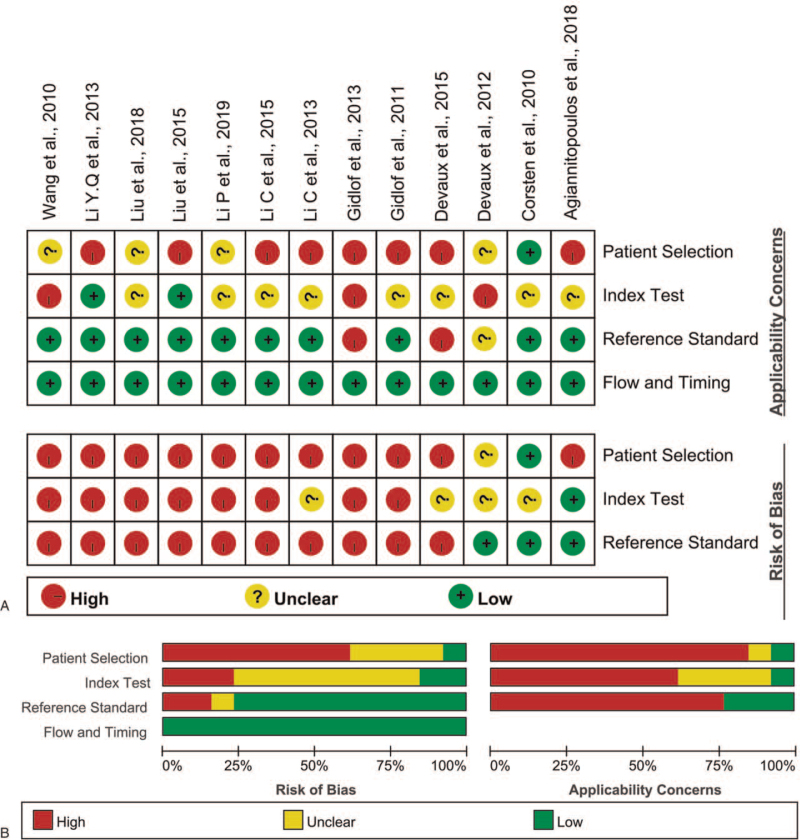
(A) Risk of bias and applicability concerns: reviewers’ judgments about each domain for each included study. (B) Bar graphs of the methodological quality assessment.

Quality assessment results of the studies reporting on miR-208 included in the meta-analysis using the QUADAS2 evaluation tool are shown in Figure 2A. Results are presented as percentages across the studies (Fig. 2B).

### 3.3 Diagnostic accuracy

**Figure 3:**
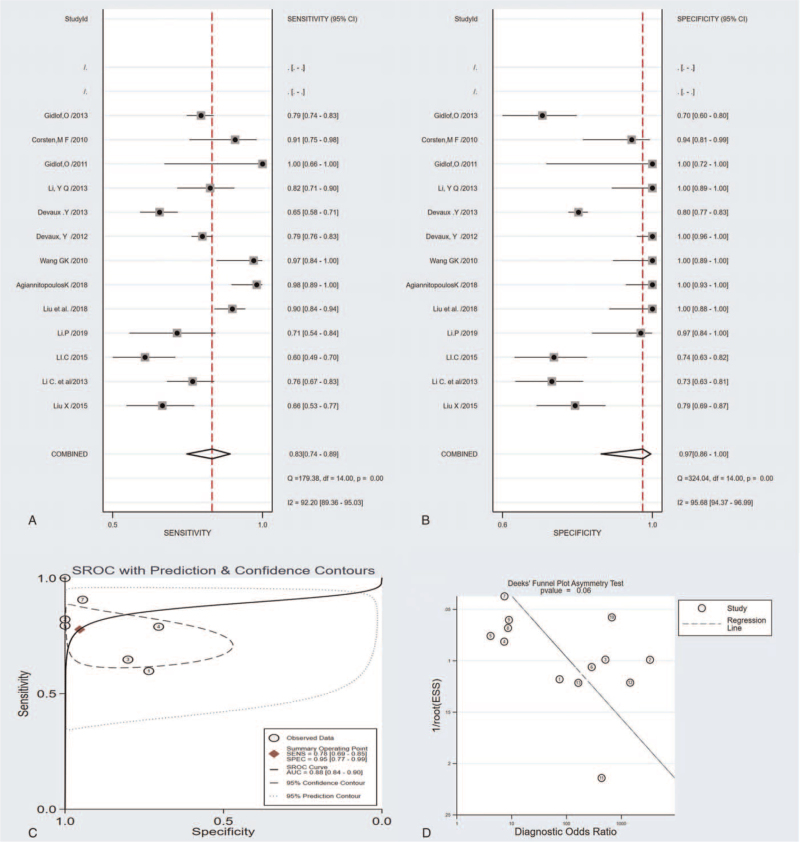
The sensitivity, specificity, summary receiver operator characteristic (SROC) curve with area under the curve (AUC), and funnel graph of the miRNA-208 family in diagnosing acute myocardial infarction. (A) Sensitivity. (B) Specificity. (C) SROC curve with AUC. (D) Funnel graph.

Sensitivity analyses were performed on the included studies according to the following factors: the following factors overall sensitivity for the circulating miR-208 was 0.83 (95% CI: 0.74–0.89), the overall specificity was 0.97 (95% CI: 0.86–1.00), the PLR was 10.28 (95% CI: 4.45–23.77), the NLR was 0.17 (95% CI: 0.10–0.31), the diagnostic OR and 95% confidence interval were 59.22 and 16.29 to 215.32, the summary receiver operator curve (SROC) analysis for the studies yielded an overall weighted area under the curve of 0.93 (95% CI: 0.91–0.95) (Fig. 3C). We conducted a subgroup analysis and the results were as follows:

1. Type of miRNA detection method (SYBR green vs TaqMan): the SROC values were 0.92 versus0.94, the pooled sensitivity and specificity were 0.82 versus 0.84 and 0.96 versus 0.99, respectively;
2. Included studies size (Sample size ≤100 versus Sample size >100): the SROC values were 0.94 versus 0.82, the pooled sensitivity and specificity were 0.77 versus0.78 and 0.88 versus 0.81, respectively;
3. Different population (Caucasia vs East Asian) the SROC values were 0.96 versus 0.91, the pooled sensitivity and specificity were 0.87 versus 0.80 and 0.98 versus 0.97, respectively;
4. Time of blood sampling (the onset of symptoms <5 h vs the onset of symptoms <24 hours) the SROC values were 0.86 versus 0.97, the pooled sensitivity and specificity were 0.75 versus 0.88 and 0.89 versus 0.99, respectively;
5. Patient characteristics (Control) (AMI/Healthy vs AMI/non-AMI) the SROC values were 0.93 versus 0.93, the pooled sensitivity and specificity were 0.83 versus 0.83 and 1.00 versus 0.93, respectively.

A summary of the sensitivity analysis results was shown in Table 2.

**Table 2:** Comparison of miR-208 among subgroups in acute myocardial infarction.

	AUC (95% CI)	Sensitivity (95% CI)	Specificity (95% CI)	PLR (95% CI)	NLR (95% CI)	DOR (95% CI)
miRNA-208 (n = 13)	0.93 (0.91–0.95)	0.83 (0.74–0.89)	0.97 (0.86–1)	31.8 (5.3–191.1)	0.18 (0.11–0.28)	179 (21–1509)
miRNA-208b (n = 7)	0.88 (0.84–0.9)	0.78 (0.69–0.85)	0.95 (0.77–0.99)	16.8 (2.8–99.5)	0.23 (0.16–0.35)	72 (9–568)
Type of miRNA detection method						
SYBR green (n = 7)	0.92 (0.89–0.94)	0.82 (0.73–0.89)	0.96 (0.81–0.99)	21.5 (3.7–126.2)	0.19 (0.12–0.3)	115 (14–934)
TaqMan (n = 6)	0.94 (0.92–0.96)	0.84 (0.66–0.93)	0.99 (0.38–1)	122 (0.5–30174.3)	0.16 (0.07–0.38)	745 (2–336073)
Included studies size						
Sample size ≤ 100 (n = 8)	0.94 (0.92–0.96)	0.77 (0.73–0.81)	0.88 (0.84–0.91)	12.75 (4.34–37.41)	0.19 (0.1–0.35)	92 (19–443)
Sample size > 100 (n = 5)	0.82 (0.73–0.89)	0.78 (0.75–0.8)	0.81 (0.78–0.83)	3.72 (2.14–6.47)	0.26 (0.17–0.39)	15.24 (6.46–35.94)
Different population						
Caucasis (n = 5)	0.96 (0.94–0.97)	0.87 (0.72–0.95)	0.98 (0.75–1)	45.2 (2.7–758.6)	0.13 (0.05–0.32)	343 (111–10396)
East Asian (n = 8)	0.91 (0.88–0.93)	0.80 (0.69–0.88)	0.97 (0.74–1)	2.92 (2.2–3.87)	0.15 (0.07–0.34)	27.76 (8.93–86.25)
Time of blood sampling						
The onset of symptoms <5 h (n = 5)	0.86 (0.82–0.88)	0.75 (0.63–0.84)	0.89 (0.67–0.98)	6.9 (1.8–26.6)	0.28 (0.17–0.48)	24 (4–150)
The onset of symptoms <24 h (n = 8)	0.97 (0.95–0.98)	0.88 (0.76–0.95)	0.99 (0.82–1)	126.1 (4–3935.4)	0.12 (0.06–0.25)	1040 (11–48534)
Diseases						
AMI/Healthy (n = 8)	0.93 (0.91–0.95)	0.83 (0.71–0.91)	1 (0.68–1)	198 (0.17–23077.9)	0.17 (0.1–0.3)	1165 (7–199641)
AMI/Non-AMI ACS (n = 5)	0.93 (0.91–0.95)	0.83 (0.69–0.92)	0.93 (0.74–0.98)	11.6 (2.6–50.6)	0.18 (0.09–0.39)	63 (8–511)

### 3.4 Heterogeneity

**Figure 4:**
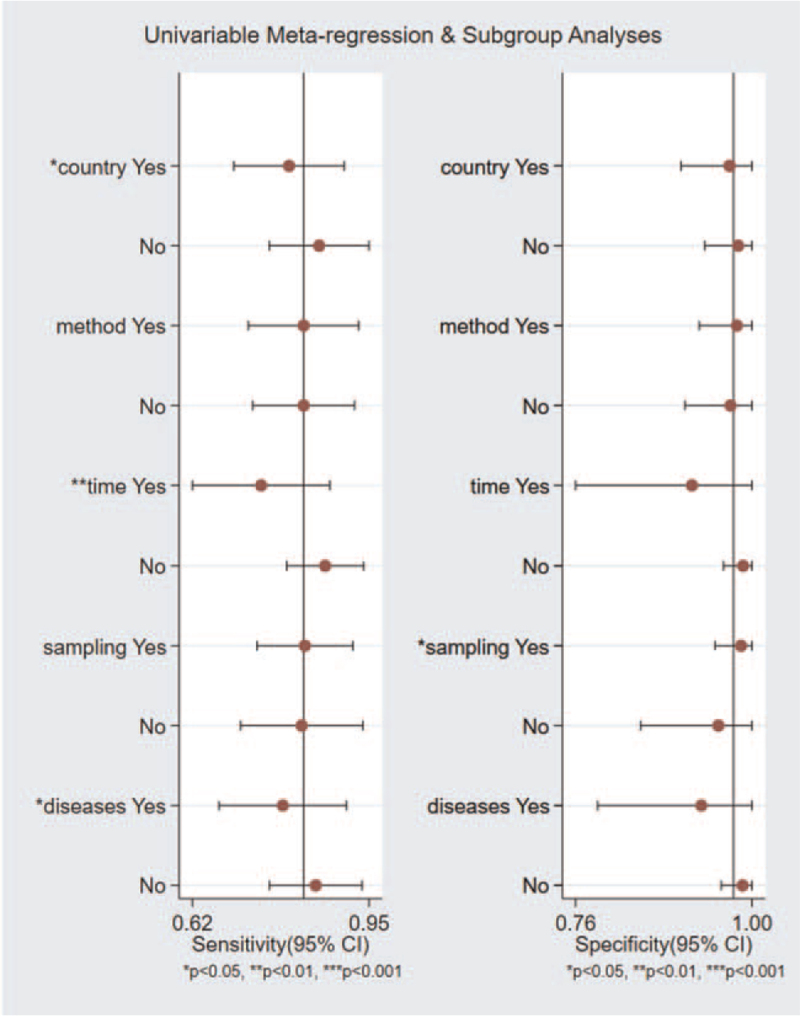
Univariable metaregression and subgroup analyses.

In order to prove the reliability of this study, we conducted a heterogeneity analysis. The result of the heterogeneity was *I*^2^ = 91% and *P* < .05. The results indicated that heterogeneity between eligible studies was observed. To assess publication bias of the included studies, we conducted a meta regression analysis to explore heterogeneity's potential sources. Subgroup analyses were performed to explore the potential source of heterogeneity. We used study covariates such as country, method, time, sampling, disease, as shown in Figure 4. Time of blood sampling was the most important source of heterogeneity; the 8 studies within 24 hours after the onset of symptoms showed a high-pooled sensitivity (0.88, 95% CI: 0.76–0.95) and specificity (0.99, 95% CI: 0.82–1.00). In contrast, the rest of the 5 studies that blood be taken within 5 hours after the onset of symptoms showed a low-pooled sensitivity (0.75, 95% CI: 0.63–0.84) and a low-pooled specificity (0.89, 95% CI: 0.67–0.98) (Table 2).

### 3.5 Publication bias

The potential publication bias in all included studies was assessed using Deeks’ funnel plot asymmetry test. The regression line's slope coefficient had a *P* value of .06; the Deeks’ test suggested publication bias may affect the summary estimates (Fig. 3D).

## 4 Discussion

### 4.1 Mir-208 as an early biomarker for the diagnosis of AMI

**Figure 5:**
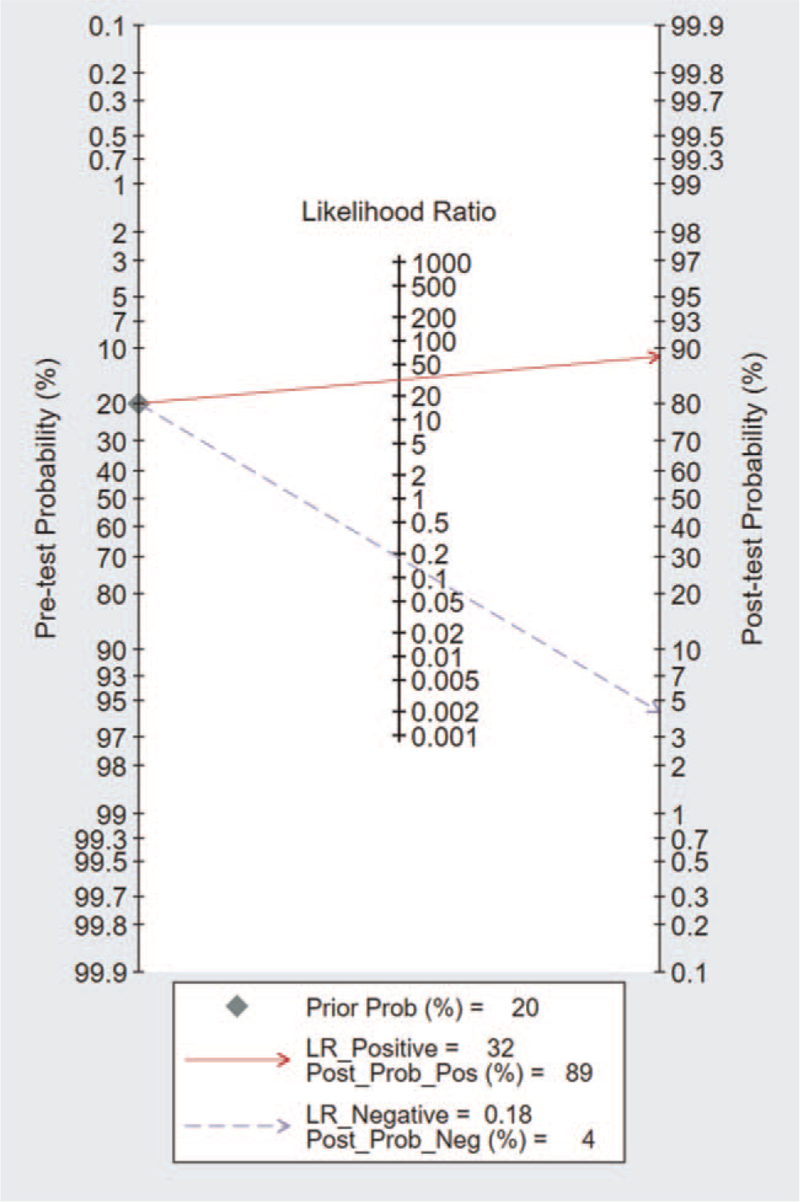
Fagan nomogram of circulating microRNA-208 for diagnosis of acute myocardial infarction.

AMI was a disease with a high mortality rate of coronary heart disease, which usually causes damage to myocardial tissue. Currently, the understanding of AMI's molecular mechanism was still limited, so we need accurate biomarkers to predict the risk of AMI.^[30]^ With the development of research techniques, some new biomarkers, such as CK-MB and troponin, have been used to detect blood. However, the role of these biomarkers in the diagnosis of early myocardial infarction was still limited.^[31–33]^ Some studies have shown that cardiac troponins and creatine kinase MB, were not effective at very early diagnosis of AMI (within 0–3 hours).^[33]^ However, the search for additional early biomarkers, especially those with different underlying molecular mechanisms, may lead to higher sensitivity and specificity in a shorter period of time. MiRNAs are involved in various fundamental biological processes,^[34]^ including observing the development of stable, circulating proliferation, differentiation, and apoptosis, which have led to a rapid increase in reports of the use of these molecular biomarkers for various diseases.^[35]^ Some studies have confirmed the diagnostic value of miR-208 for AMI.^[36]^ MiR-208 can influence the proliferation of the myocardial cells; these were consistent with the most recent research results. MiR-208 was a kind of heart specificity of microRNAs. Coding in major histocompatibility complex (MHC) genes was an active adjustment factor of the MHC gene expression after myocardial infarction, and miR-208 microbubble was released into the peripheral circulation of blood in the form of myocardial cell apoptosis and inhibits the effective molecular targets NLK expression. Besides, reducing the reactive oxygen species in the miR-208 by influencing the myocardial cells to increase myocardial apoptosis.^[26,37,38]^ Although there have been many articles on the diagnostic value of miR-208 for AMI, the conclusions are still controversial. The accuracy and efficiency of these studies are primarily affected by the sample size. Therefore, this paper aims to use meta-analysis as a powerful and useful tool to expand the sample size and improve stability. A total of 13 pieces of literature were included in this study. The area under the SROC curve for miR-208 was 0.93 [95% CI: 0.91–0.95] (Fig. 3). The results of the current study proved that circulating miR-208 has good sensitivity and specificity for differentiating AMI from non-AMI (0.83 and 0.97) respectively. These results of sensitivity and specificity are similar or even better than those reported in a previous study.^[39]^ To be more clinically informative in our results, the pooled LRs were used to estimate post-test probabilities. A PLR of 31.8 implies that a person with AMI has about 32 times more likely to be miR-208-positive than a non-AMI person. The NLR of 0.18 suggested that a person with AMI was 18% if the circulating miR-208 was negative (Fig. 5). However, this article also has its limitations. First of all, the articles retrieval were mainly written in English, making the loss of some high-quality articles written in other languages. The sample size was reduced; Secondly, articles used in the study are published mainly in the developed countries, which was publication bias; third, the testing standards were not unified with the miRNA-208 detection time. The detection process is not entirely standardized, so the miR-208 clinical value for AMI diagnosis remains unclear. Besides, the range of time to collect the specimens was vast.

Although miR-208 had been identified as an early biomarker of AMI in this study (<24 hours), in order to verify that Mir-208 was an earlier biomarker of AMI (<5 hours). Therefore, we were only able to subdivide the studies into 2 main groups:

1. Measurement within 5 hours
2. Measurement within 24 hours.

This result was different from previous studies. The measured data within 24 hours has a relatively higher AUC value; the Area under the SROC Curve was 0.97 (95% CI: 0.95–0.98) (Table 2). However, there were a considerable individual variation in each study. The AUC value obtained within 5 hours was 0.86 (95% CI: 0.82–0.88) may suggest miR-208 as a diagnostic tool in a specific population. It was necessary to increase the sample size for further studies and repeated time measurements.

### 4.2 MiR-208 in healthy and unhealthy controls

An important methodological issue in investigating the potential of miR-208 as a diagnostic marker for AMI was control selection. Early reperfusion, usually through percutaneous coronary intervention (PCI), was a primary factor in the prognosis and clinical outcome of AMI.^[40,41]^ Severe neointimal proliferation and hyperplasia, vascular remodeling, increased vascular smooth muscle cell proliferation and migration, and chronic inflammation after the coronary stent implantation procedure are the main causes of restenosis.^[10]^ MiR-208 had a higher AUC value in healthy controls and patients with acute chest pain and the mixed population. When used healthy controls, the total AUC of miR-208 was 0.93 (0.91–0.95); the total AUC of miR-208 was 0.93 (95% CI: 0.91–0.95) in the unhealthy control group. We may safely conclude that miR-208 distinguishes between AMI patients with acute chest pain and healthy controls are useful. It was reasonable to assume that the miR-208 could cause any typical symptoms in patients with the diagnosis effect of pain. MiR-208 in diagnosing patients with specific cardiac symptoms, potential diagnostic pain still played better in early treatment. There was limited medical literature on improving the treatment efficiency on this subject, this speculation requires further evaluation in prospective studies.

### 4.3 Mir-208 as a biomarker that distinguishes between STEMI and NSTEMI

**Figure 6:**
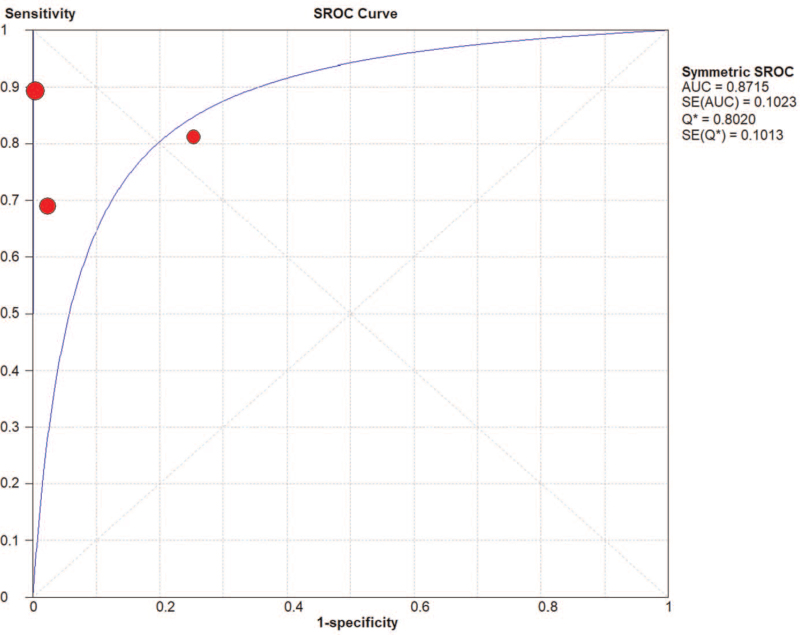
The area under the SROC curve for NSTEMI.

**Figure 7:**
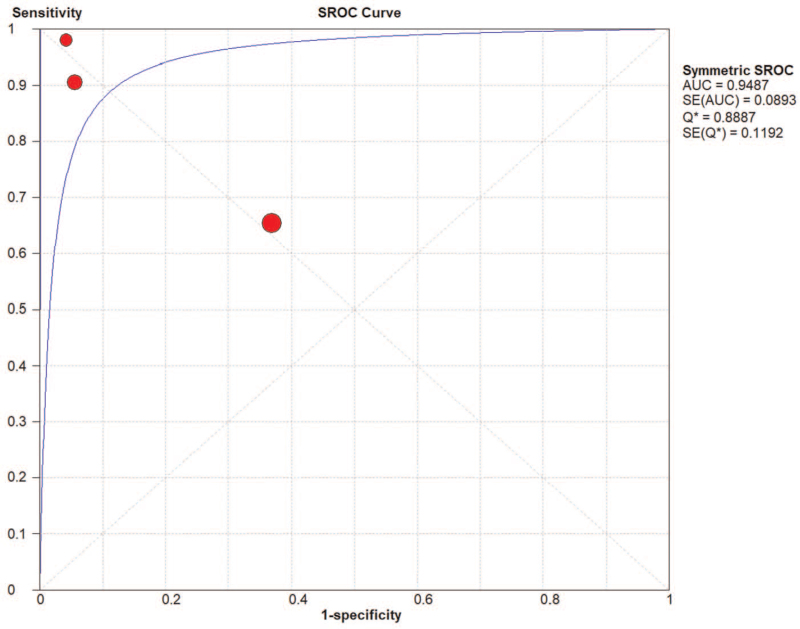
The area under the SROC curve for STEMI.

**Figure 8:**
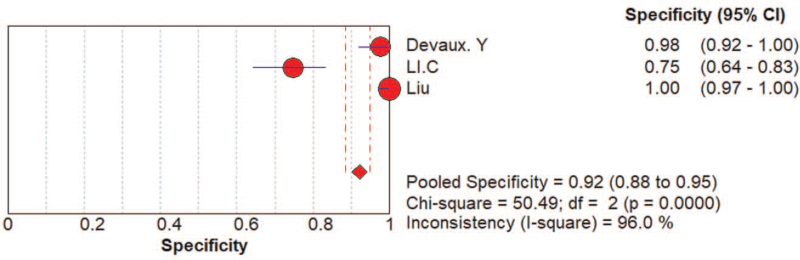
The specificity of the miRNA-208 family in the diagnosis of NSTEMI patients.

**Figure 9:**
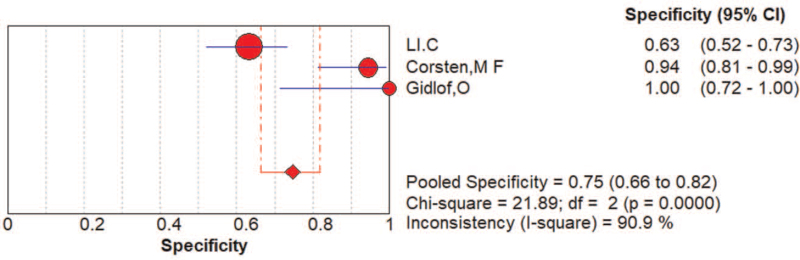
The specificity of the miRNA-208 family in the diagnosis of STEMI patients.

AMI was a common and fatal medical emergency at present. It was vital to acquire a fast and accurate AMI diagnosis and improve the detection method for high-risk patients. Currently used circulating biomarkers such as cardiac troponins and creatine kinase MB act as sensitive and specific tests for myocardial damage, yet, they may be negative early in the process of ischemia. Their increase in the setting of STEMI, a process that nearly always results from coronary plaque rupture and thrombosis formation, was usually reflective of the extent of the infarct and approximates the mass of cardiomyocytes that damaged in the process of AMI. In the setting of NSTEMI, increases in different biomarkers may be suggestive of a specific underlying pathophysiology, although data was limited on such associations. Consequently, it was of great importance to develop new and improved risk stratification tools that will allow clinicians to recognize high risk NSTEMI patients, and specifically those with occult TO, as early as possible. In this study, we analyze the value of the diagnosis of miR-208 and divide patients into STEMI.^[13,16,17]^ And NSTEMI patients,^[13,18]^ the pooled AUC of NSTEMI patients was 0.87 (Fig. 6). The pooled AUC of STEMI patients was 0.95 (Fig. 7). The specificity (0.92, 95% CI: 0.88–0.95) of 3 studies that reviewed NSTEMI controls was higher than the specificity (0.75, 95% CI: 0.66–0.82) in 3 studies performed in STEMI controls (Figs. 8 and 9). However, Devaux et al ^[18]^ and Devaux et al ^[21]^ regarded that the elevated level of miR-208 in STEMI was higher than in NSTEMI. In contrast, the results reported by Li ^[24]^ shows the elevated level of miR-208 in NSTEMI was higher than in STEMI. We assume that different modes of the miR-208 increase may characterize a particular entity's myocardial injury. However, there was limited literature about meta-analysis and the study of specific STEMI and NSTEMI data effective. Moreover, there was no report indicating the potential causes of the myocardial injury; it was unclear whether these possible causes can show the different modes of mir-208 rise; it may had diagnostic importance before coronary angiography. To fully evaluate the miR-208 diagnosis of potential in this respect, we need to increase the sample size. Further research would be required. Therefore, miR-208 in NSTEMI plasma concentration and whether the differences between the dynamics can determine the underlying pathophysiology of difference, and whether these differences can accurately stratify risk groups are discriminated.

## 5 Clinical significance and innovation

In this paper, all the articles published in recent years on the value of miR-208 in the diagnosis of AMI were included, so the results of this study are more reliable and convincing. In addition, we strictly considered the precise setting of specific micrornas in diagnosis and evaluation. In this study, we found that miR-208 could not only distinguish between healthy and AMI patients, but also had some value in AMI in chest pain patients. This provides reference for early diagnosis of the disease and accurate treatment plan. Provide time to develop targeted treatment plans. We also investigated the expression of Mir-208 in STEMI and NSTEMI, and we found that AUC was slightly higher in STEMI patients, but due to the small sample size, it could only provide predictive directions for clinical treatment.

## 6 Conclusion

In conclusion, there was significant heterogeneity in the study. More clearly grouped samples were needed to corroborate the analysis. The inclusion of only English articles may cause vital research to be neglected and lead to publication bias. Even so, we found that miR-208 does have the diagnostic capability (a total AUC of 0.93), although it was still inferior to existing modalities.^[42,43]^ Simultaneously, we suggest that it may be used to detect specific types of heart injury and differentiate between AMI and non-acute myocardial infarction patients with chest pain. MiR-208 also has high diagnostic value in both STEMI and NSTEMI.

Further studies are needed to determine the clinical applicability of miR-208 in these different conditions. Finally, we emphasize that miR-208 may still highly expressed in AMI, and miR-208 has the potential to guide treatment more accurately. Therefore, further studies to formulate a standardized diagnostic criterion and identify the optimal cut-off values are required.

## Author contributions

**Conceptualization:** Jia Wang.

**Data curation:** Jia Wang, Liwenjing Xu, Lu Tian, Qiyu Sun.

**Formal analysis:** Jia Wang.

**Project administration:** Qiyu Sun.

**Software:** Jia Wang.

**Supervision:** Qiyu Sun.

**Validation:** Jia Wang, Liwenjing Xu, Lu Tian, Qiyu Sun.

**Visualization:** Jia Wang, Liwenjing Xu, Lu Tian, Qiyu Sun.

**Writing – original draft:** Jia Wang.

**Writing – review & editing:** Jia Wang, Qiyu Sun.
